# The severity of acute hypoxaemia determines distinct changes in intracortical and spinal neural circuits

**DOI:** 10.1113/EP091224

**Published:** 2023-08-07

**Authors:** Daniel J. McKeown, Glenn M. Stewart, Justin J. Kavanagh

**Affiliations:** ^1^ Neural Control of Movement Laboratory Menzies Health Institute Queensland Griffith University Gold Coast Queensland Australia; ^2^ Department of Psychology Faculty of Society and Design Bond University Gold Coast Queensland Australia; ^3^ Menzies Health Institute Queensland Griffith University Gold Coast Queensland Australia; ^4^ Allied Health Research Collaborative The Prince Charles Hospital Brisbane Queensland Australia; ^5^ Charles Perkins Centre The University of Sydney Sydney New South Wales Australia

**Keywords:** high altitude, neuromodulation, transcranial magnetic stimulation

## Abstract

The purpose of this study was to examine how two common methods of continuous hypoxaemia impact the activity of intracortical circuits responsible for inhibition and facilitation of motor output, and spinal excitability. Ten participants were exposed to 2 h of hypoxaemia at 0.13 fraction of inspired oxygen ($F_{IO_{2}}$ clamping protocol) and 80% of peripheral capillary oxygen saturation ($S_{pO_{2}}$ clamping protocol) using a simulating altitude device on two visits separated by a week. Using transcranial magnetic and peripheral nerve stimulation, unconditioned motor evoked potential (MEP) area, short‐interval intracortical inhibition (SICI) and intracortical facilitation (ICF), and F‐wave persistence and area were assessed in the first dorsal interosseous (FDI) muscle before titration, after 1 and 2 h of hypoxic exposure, and at reoxygenation. The clamping protocols resulted in differing reductions in $S_{pO_{2}}$ by 2 h ($S_{pO_{2}}$ clamping protocol: 81.9 ± 1.3%, $F_{IO_{2}}$ clamping protocol: 90.6 ± 2.5%). Although unconditioned MEP peak to peak amplitude and area did not differ between the protocols, SICI during $F_{IO_{2}}$ clamping was significantly lower at 2 h compared to $S_{pO_{2}}$ clamping (*P* = 0.011) and baseline (*P* < 0.001), whereas ICF was higher throughout the $F_{IO_{2}}$ clamping compared to $S_{pO_{2}}$ clamping (*P* = 0.005). Furthermore, a negative correlation between SICI and $S_{pO_{2}}$ (*r*_rm_ = −0.56, *P* = 0.002) and a positive correlation between ICF and $S_{pO_{2}}$ (*r*_rm_ = 0.69, *P* = 0.001) were determined, where greater reductions in $S_{pO_{2}}$ correlated with less inhibition and less facilitation of MEP responses. Although F‐wave area progressively increased similarly throughout the protocols (*P* = 0.037), persistence of responses was reduced at 2 h and reoxygenation (*P* < 0.01) during the $S_{pO_{2}}$ clamping protocol compared to the $F_{IO_{2}}$ clamping protocol. After 2 h of hypoxic exposure, there is a reduction in the activity of intracortical circuits responsible for inhibiting motor output, as well as excitability of spinal motoneurones. However, these effects can be influenced by other physiological responses to hypoxia (i.e., hyperventilation and hypocapnia).

## Abstract (highlights)

- **What is the central question of this study?**
  How do two common methods of acute hypoxic exposure influence the excitability of intracortical networks and spinal circuits responsible for motor output?
- **What is the main finding and its importance?**
  The excitability of spinal circuits and intracortical networks responsible for inhibition of motor output was reduced during severe acute exposure to hypoxia at 2 h, but this was not seen during less severe exposure. This provides insight into the potential cause of variance seen in motor evoked potential responses to transcranial magnetic stimulation (corticospinal excitability measures) when exposed to hypoxia.

## 1 INTRODUCTION

Exposure to a low oxygen environment presents a significant challenge to the central nervous system (CNS). A decreased partial pressure of inspired oxygen ($P_{iO_{2}}$) can reduce arterial partial pressure of oxygen ($P_{aO_{2}}$) and increase the stimulation of arterial chemoreceptors and mechanoreceptors located in carotid and aortic bodies and the lungs (Eyzaguirre & Koyano, 1965; Rahn & Otis, 1949). In response, respiratory centres located within the brainstem increase phrenic and sympathetic output, and as a consequence, hypoxic hyperventilation, hypoxic pulmonary vasoconstriction and cerebral vasodilatation ensue. This is in an effort to mitigate the adverse consequences of hypoxaemia and the limited oxygen that is available in active tissues (i.e., hypoxia; Amann et al., 2013). Although cerebrovascular adaptations occur in an effort to maintain oxygenation of neural tissue, severe hypoxaemia often results in cerebral hypoxia and impaired activity of motor pathways (Barnard et al., 1987; Fuller et al., 2001; Gonzalez‐Alonso et al., 2004; Goodall et al., 2010; Moudgil et al., 2005).

As acute hypoxaemia (exposures <2 h) may have little effect on the excitability of α‐motoneurones directly (Christiansen et al., 2018; Eccles et al., 1966; Ruggiero et al., 2018; Szubski et al., 2006), there is some suggestion that hypoxia‐related changes in motor activity originate presynaptically to α‐motoneurones in the motor cortex and between corticospinal‐motoneuronal synapses (Christiansen et al., 2018; Goodall et al., 2012, 2010). However, the impact that hypoxia has on the output of motor pathways is complex and at times divergent. Notably, during acute continuous hypoxaemia, transcranial magnetic stimulation (TMS)‐evoked motor evoked potentials (MEPs) arising from the primary motor cortex can increase (Goodall et al., 2012; McKeown et al., 2020; Rupp et al., 2012), decrease (McKeown et al., 2021) or remain unchanged (Goodall et al., 2010; McKeown et al., 2020, 2022; Millet et al., 2012; Szubski et al., 2006) when performing isometric contractions of the first dorsal interosseous (FDI), biceps brachii and quadriceps. These equivocal responses are likely due to variation in contraction tasks, hypoxaemia modalities and the hypoxic severity experienced by neural tissue ($S_{pO_{2}}$; Rojas‐Camayo et al., 2018; Ruggiero et al., 2022).

What is yet to be considered is the influence hypoxia has on other cortical neural circuitry. Although corticospinal neurones are responsible for propagating motor output to activate α‐motoneurones, interactions from other cortical presynaptic neural circuits are able to modulate this output resulting in either facilitation (glutamatergic circuits) or inhibition (GABAergic circuits) of the MEP (Hanajima et al., 1998; Kujirai et al., 1993; Ziemann et al., 1995). In vitro rat and primate models have demonstrated that these neural circuits are particularly vulnerable and are less excitable when hypoxic (Romijn, 1989; Sloper et al., 1980). However, whether this is consistent in the intact human nervous system is poorly understood.

In the few studies that have used paired‐pulse TMS to assess intracortical circuits, GABAergic circuits responsible for inhibition (signified by short‐interval intracortical inhibition; SICI) and glutamatergic circuits responsible for facilitation (signified by intracortical facilitation; ICF) remain unchanged during short hypoxaemic exposures (30 min; Szubski et al., 2006). However, this adaptation may be transient as longer durations of exposure reduce GABAergic but not glutamatergic circuit activity (Miscio et al., 2009). Considering this, the impact of hypoxia on these intracortical networks may be circuit‐specific, as well as time‐dependent in a similar manner to corticospinal motoneurones (Romijn, 1989; Rupp et al., 2012; Sloper et al., 1980). Furthermore, as the experimental control of hypoxic exposure has typically involved the titration of the fraction of inspired oxygen ($F_{IO_{2}}$) in air (i.e., hypoxic exposure based on a set $F_{IO_{2}}$) and not on the change in the resultant hypoxaemia (e.g., hypoxic exposure based on a set $S_{pO_{2}}$), it is unclear if this difference in methodology contributes to the variance in MEP findings.

The purpose of this study was to examine how two acute continuous hypoxia protocols impact GABAergic and glutamatergic circuits responsible for inhibition (SICI) and facilitation (ICF) of corticospinal excitability, respectively, as well as spinal excitability. This was achieved by employing paired‐pulse TMS and ulnar nerve stimulation to assess EMG responses in the unfatigued FDI throughout two commonly used methods of hypoxaemia exposure. On separate occasions, the $F_{IO_{2}}$ and $S_{pO_{2}}$ of participants were titrated to 0.13 and 80%, respectively, to determine if neuromuscular responses varied between methods. Participants were exposed to each continuous hypoxic stimulus for a period of 2 h during two separate visits. It was hypothesised that the degree of GABAergic and glutamatergic activity on motor output would reduce throughout the hypoxaemic exposures, while spinal excitability would remain consistent.

## 2 METHODS

### 2.1 Ethical approval

Each participant provided written and witnessed informed consent prior to undertaking testing. All experimental procedures were approved by the Griffith University Human Research Ethics Committee (reference number: 2022/262). The study conformed to the standards set by the *Declaration of Helsinki*, except for registration in a database.

### 2.2 Participants

Ten heathy individuals (28 ± 5 years, 2 female) volunteered to participate in the current study. All participants completed a medical history questionnaire prior to testing. This medical history questionnaire has been used in previous hypoxia investigations of our laboratory and includes exclusion criteria specific to reduced blood oxygenation, magnetic stimulation and electrical stimulation (McKeown et al., 2020, 2019, 2021, 2022). Participants were instructed to refrain from moderate‐to‐high intensity exercise for 12 h before testing and were not permitted to take CNS medications or any form of stimulant or depressant such as caffeine or alcohol. Five participants had not been exposed to a hypoxic stimulus in the previous 6 months and five participants had never been exposed to a hypoxic stimulus. Power calculations were performed with G*Power software (v3.1.9.4) based on ICF and SICI responses previously observed in hypoxia (Miscio et al., 2009). To achieve a statistical power of 0.8 at an α level of 0.05 and effect size of 0.8, it was determined that 10 participants were required for this study.

### 2.3 Experimental design

The current study was a two‐way cross‐over design where participants attended two testing sessions that were separated by at least 1 week. For safety reasons this was a single‐blind study, as an investigator in the room needed knowledge of the intervention and monitored $S_{pO_{2}}$ throughout the testing sessions. In one session, an intervention was employed where $F_{IO_{2}}$ was clamped at 0.13 ($F_{IO_{2}}$ clamping protocol) and in the other session, an intervention was employed where the $F_{IO_{2}}$ was periodically adjusted to maintain a desired $S_{pO_{2}}$ at ∼80% ($S_{pO_{2}}$ clamping protocol). The administration of the interventions was counterbalanced to avoid order effects, where half of the participants underwent the $F_{IO_{2}}$ clamping protocol, and half of the participants underwent the $S_{pO_{2}}$ clamping protocol in their first session.

### 2.4 Instrumentation

#### 2.4.1 Hypoxia intervention

An altitude simulator (ATS200HP, Altitude Training Systems, Blacktown, NSW, Australia) and a sealed facemask was used to induce hypoxaemia and the resultant tissue hypoxia. Participants were required to wear the facemask during baseline and reoxygenation (normoxia) measures where $F_{IO_{2}}$ was held stable at 0.204. Given that sensitivity to a reduced $F_{IO_{2}}$ is variable between individuals (Rojas‐Camayo et al., 2018), a titration phase was implemented over a 15 min period. For the $F_{IO_{2}}$ clamping protocol, $F_{IO_{2}}$ was reduced at a rate of 0.01 every 1.5 min until 0.13 $F_{IO_{2}}$ was reached. For the $S_{pO_{2}}$ clamping protocol, $F_{IO_{2}}$ was reduced at the same rate until 80% $S_{pO_{2}}$ was reached. Following the titration period, participants remained in the hypoxia/hypoxaemia environment for 2 h. The severity and duration of hypoxaemia exposure was chosen based on previous research from our lab that has documented hypoxaemia‐induced changes in neuromuscular function at 80% $S_{pO_{2}}$ for 2 h (McKeown et al., 2020, 2019, 2021, 2022), while the $F_{IO_{2}}$ target was chosen as these exposures initially result in similar hypoxaemia to our $S_{pO_{2}}$ clamping protocol. These hypoxic targets correspond to approximately 4000 m above sea level (Luks & Swenson, 2011). Heart rate (derived from pulse pressure of the pulse oximeter) and $S_{pO_{2}}$ were monitored continuously using a pulse oximeter (Model 7500, Nonin Medical Inc., Plymouth, MN, USA) attached to the middle finger of the left hand. Participants were carefully monitored for symptoms of acute mountain sickness (AMS), including dizziness, headaches, sleepiness and nausea. Participants scored their experience of each individual symptom as 0 (absent), 1 (mild), 2 (moderate) or 3 (severe). Symptoms were then calculated as a cumulative score to represent the severity of AMS symptoms experienced by a participant at a time point. The Lake Louise AMS survey (Roach et al., 2018) was employed before titration (baseline), immediately after titration (post‐titration), 1 and 2 h after the titration phase, and at reoxygenation (immediate removal of face mask and exposure to ambient air).

#### 2.4.2 Electromyography

Participants sat comfortably in a chair where their right forearm was supported on a bench with their hand pronated in a custom‐designed hand plate. The wrist and digits 3–5 were secured to the hand plate with Velcro straps to prevent unwanted movement artefact. This allowed isolated finger abductions of the second digit. Surface electromyography (EMG) was recorded from the FDI by attaching circular 24 mm Ag/AgCl electrodes (Kendall Arbo) in a bipolar muscle belly–tendon arrangement (20 mm inter‐electrode distance). A ground electrode was placed on the skin overlying the distal head of the ulna on the ipsilateral limb. EMG signals were sampled at 2000 Hz using a 16‐bit analog‐to‐digital converter (CED 1401; Cambridge Electronic Design, Cambridge, UK) and Signal software (version 7.06; Cambridge Electronic Design). EMG signals were amplified (×300) and bandpass filtered (20–1000 Hz) using a CED 1902 amplifier (Cambridge Electronic Design).

#### 2.4.3 Ulnar nerve stimulation

The ulnar nerve of the right limb was stimulated at rest using single supramaximal pulses of 100 μs duration via a constant current stimulator (DS7AH, Digitimer Ltd, Welwyn Garden City, UK). A surface anode was positioned 3 cm proximal to the wrist and a surface cathode was positioned 7 cm proximal to the wrist. Optimal positioning of the surface cathode and anode was determined by using a two‐prong stimulation pen (at a stimulus intensity of 20 mA) and was based on the highest elicited M‐wave response. The purpose of ulnar stimulation was to measure the maximal compound action potential (*M*_max_) and F‐wave responses of the FDI, which was elicited by increasing the stimulus intensity until a plateau in the peak‐to‐peak EMG amplitude was reached. The stimulus intensity was then set to 50% above the current that elicited *M*_max_ ($F_{IO_{2}}$ clamping protocol: 128 ± 60 mA, 30–220 mA; $S_{pO_{2}}$ clamping protocol: 133 ± 62 mA, 30–220 mA). Student's *t‐*test determined there were no significant differences in stimulus intensity between the protocols (*P* = 0.11).

#### 2.4.4 Transcranial magnetic stimulation

Single and paired pulse TMS (Magstim BiStim 200^2^ unit, Magstim Co., Whitland, UK) paradigms were used throughout the protocols to elicit MEP responses in the FDI of the right limb. A 70‐mm double‐coil was held in a postero‐lateral position with the handle orientated laterally away from the midsagittal line of the cortex (∼45°). The coil position that elicited the largest MEP responses in the FDI was marked and maintained during stimulations in both protocols. The active motor threshold (AMT) was determined by finding the lowest stimulation intensity needed to elicit a MEP response of >50 μV in at least 5 of 10 trials when performing a subtle muscle contraction (Ziemann et al., 2015). The AMT was defined before each protocol during baseline measurements and was maintained throughout the 2 h duration of hypoxaemia. Student's *t‐*test revealed that there were no differences in the TMS intensity used to measure AMT between protocols ($F_{IO_{2}}$ clamping protocol: 38 ± 7%, 30–57%; $S_{pO_{2}}$ clamping protocol: 38 ± 7%, 31–58%; *P* = 0.87). During paired‐pulse TMS, a single test stimulus was elicited at a stimulator intensity of 120% AMT to produce unconditioned MEPs. A conditioning stimulus followed by a test stimulus was applied to assess changes in glutamatergic (ICF) and GABAergic (SICI) intracortical circuits. For ICF, the subthreshold 90% AMT conditioning stimulus preceded the supramaximal 120% AMT test stimulus by 15 ms. For SICI, the subthreshold 80% AMT conditioning stimulus preceded the supramaximal 120% AMT test stimulus by 3 ms (Rothwell et al., 2021).

### 2.5 Experimental procedures

Participants visited the laboratory on two occasions, where they performed the $F_{IO_{2}}$ clamping protocol or the $S_{pO_{2}}$ clamping protocol on their initial visit. Neurophysiological measurements were obtained immediately before the 15 min titration phase (baseline), 1 and 2 h following the titration phase, and at reoxygenation. At baseline, the stimulus intensity needed to elicit *M*_max_ and AMT of each participant was determined. At each time point, *M*_max_ and F‐wave responses were measured via electrical stimulation of the ulnar nerve and were extracted from the FDI EMG. In total, four *M*_max_ and 30 F‐wave responses were collected for each participant at each point in time. Following this, single and paired‐pulse TMS was used to elicit unconditioned MEP, ICF and SICI responses. Twenty responses of each measurement were collected in a randomised order for each participant at each point in time. Intraclass correlations were calculated at baseline, which indicated that there was excellent within‐participant repeatability for each neurophysiological measurement (>0.74; Table 1).

**TABLE 1:** The mean intraclass correlations (ICCs) for within‐subject repeatability of each neurophysiology measurement at baseline.

	ICC	
	$F_{IO_{2}}$ clamping protocol (*n* = 10)	$S_{pO_{2}}$ clamping protocol (*n* = 10)
*M*_max_	0.99	0.99
F waves	0.76	0.82
Unconditioned MEP	0.86	0.79
SICI MEP	0.83	0.82
ICF MEP	0.86	0.74

$F_{IO_{2}}$, fraction of inspired oxygenation; ICF, intracortical facilitation; *M*_max_, maximal compound action potential; MEP, motor evoked potential; $S_{pO_{2}}$, peripheral capillary oxygen saturation; SICI, short‐interval intracortical inhibition.

### 2.6 Data analysis

All electrophysiology data were analysed offline using Signal (version 7.06, Cambridge Electronic Design). Scores from the Lake Louise AMS survey are reported as a cumulative score. *M*_max_ area was calculated from the onset of the first deflection to the offset of the final deflection of the EMG following the stimulation artefact. M‐wave responses were ensemble‐averaged to provide a single *M*_max_ area for each time point. To measure F‐wave responses, a high‐pass, fourth order Butterworth filter with a cut‐off frequency of 220 Hz was applied offline. High‐pass filtering ensured accurate assessment of the F‐wave response without interferences from the preceding M‐wave (Kavanagh et al., 2019; Khan et al., 2012). F‐wave responses were included in analysis if the EMG response emerged at a latency greater than 25 ms and had an amplitude greater than 0.025 mV above baseline. The area of each F‐wave was normalised to the preceding *M*_max_ during each contraction. The persistence of F‐wave responses was calculated as the percentage of F‐waves identified out of the 30 ulnar nerve stimulations applied. The area of unconditioned and conditioned MEP responses during paired‐pulse TMS were calculated as the onset of the first deflection to the offset of the final deflection of the EMG following the stimulation artefact. MEP responses were ensemble‐averaged to provide a single unconditioned MEP area, ICF area and SICI area for each time point. Responses were then normalised to the area of the *M*_max_. ICF and SICI were normalised to the area of the unconditioned MEP. Normalised SICI and ICF MEP responses are expressed as changes from baseline measurements where a value >100% indicates facilitation and a larger MEP response and a value <100% indicates inhibition and a smaller MEP response.

### 2.7 Statistical analysis

Normality of data was assessed using the Shapiro–Wilk test. Greenhouse–Geisser corrections were applied when assumptions of sphericity were violated. Differences between baseline measurements in both protocols were assessed using Student's paired *t*‐test. Restricted maximum likelihood mixed‐effects models were employed to examine a main effect of protocol ($F_{IO_{2}}$ clamping protocol vs. $S_{pO_{2}}$ clamping protocol) and a main effect of time (baseline, post‐titration, 1 and 2 h following titration, and reoxygenation). Where a significant main effect of time was identified, Šídák's multiple comparisons test was used to identify at which time point each variable differed from baseline measurements. Protocol by time interaction effects were also examined for each dependent variable, where Šídák's multiple comparisons test was used to identify the time point(s) where the protocols differed from each other. Correlative relationships for changes in SICI and ICF responses in relation to changes in $S_{pO_{2}}$ were assessed using repeated measures correlation coefficients (*rmcorr* package; Bakdash & Marusich, 2017). As two different levels of hypoxaemia resulted between the two clamping protocols, the correlations include both a $F_{IO_{2}}$ clamping protocol ICF and SICI data point and a $S_{pO_{2}}$ clamping protocol ICF and SICI data point for each participant. Repeated measures correlation coefficients were used, as this model accounts for the non‐independence among responses using analysis of covariance (ANCOVA). The model provides the best linear fit for each participant using parallel regression lines with varying intercepts. This model has been recently used in neurophysiological research where multiple responses are recorded from participants (Orssatto et al., 2023). All statistical procedures were performed using GraphPad Prism (v.9; GraphPad Software, San Diego, CA, USA) with α levels set at <0.05. Repeated measures correlations were analysed in R with α levels set at <0.05. Data in figures are presented as group means and error bars represent the standard deviation.

## 3 RESULTS

### 3.1 $F_{IO_{2}}$ and $S_{pO_{2}}$ responses for each intervention

**FIGURE 1:**
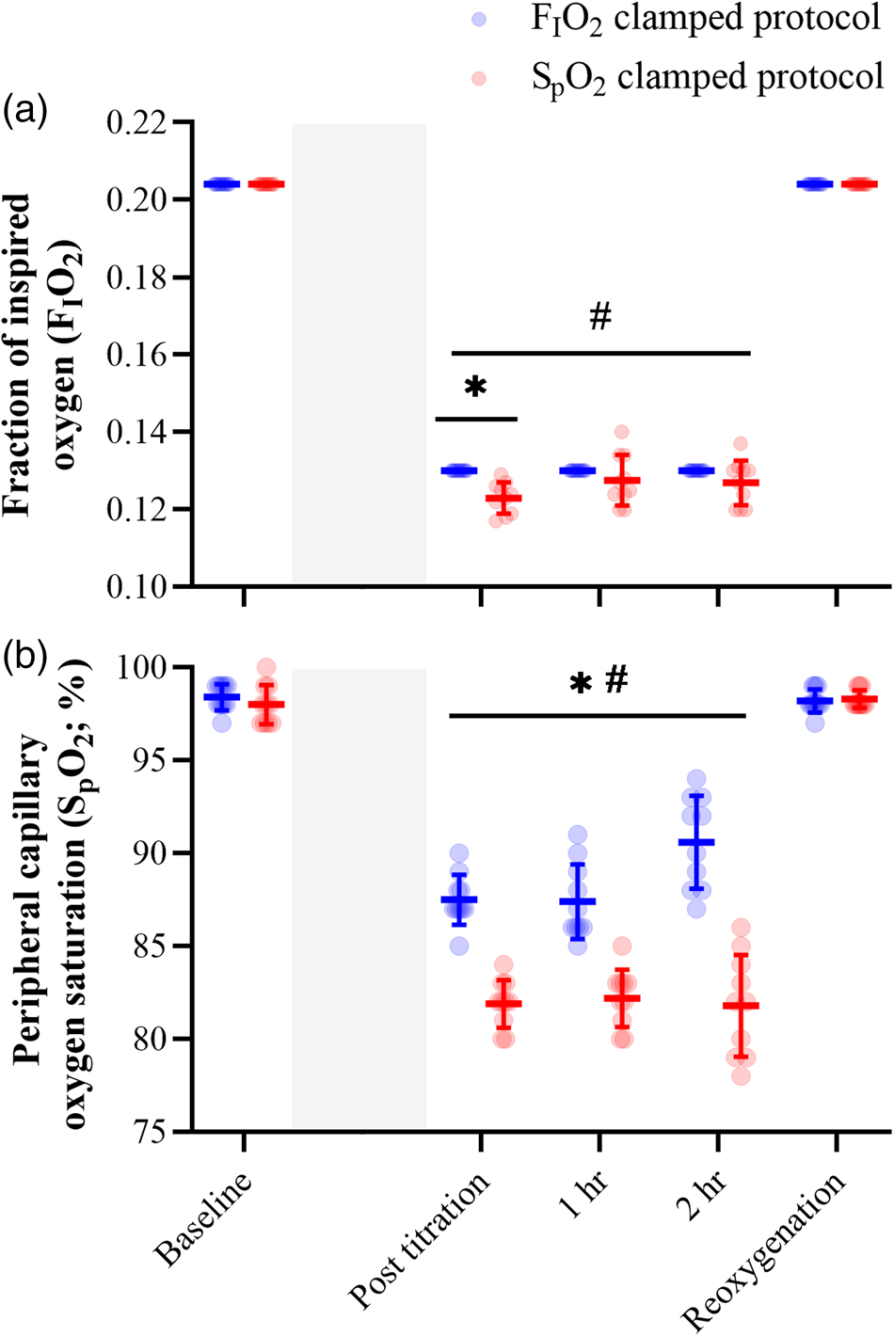
Fraction of inspired oxygen (a) and peripheral capillary oxygen saturation (b) responses were monitored throughout both interventions. Hypoxaemia was induced via a protocol that clamped $F_{IO_{2}}$ and a protocol that clamped $S_{pO_{2}}$. $F_{IO_{2}}$ and $S_{pO_{2}}$ measurements were made at baseline prior to each protocol, and then immediately post‐titration, 1 h post‐titration, 2 h post‐titration, and at reoxygenation after the removal of the hypoxic stimulus using an oxygen analyser and finger pulse oximeter, respectively. Shaded area represents titration phase. Blue represents the $F_{IO_{2}}$ clamping protocol while red represents the $S_{pO_{2}}$ clamping protocol. #Significant *post hoc* analysis from main effect of time for $F_{IO_{2}}$ and $S_{pO_{2}}$ (*P* < 0.0001). *Significant effect of clamping arising from *post hoc* analysis of the interaction effect for $F_{IO_{2}}$ (*P* = 0.001) and $S_{pO_{2}}$ (*P* < 0.0001). Data are presented as the group mean and error bars represent the standard deviation (*n* = 10 participants for all variables).

The $F_{IO_{2}}$ and $S_{pO_{2}}$ level of each participant was monitored throughout both protocols. A main effect of time was detected for $F_{IO_{2}}$ (*F*_2.16,38.84_ = 5406, *P* < 0.0001) and $S_{pO_{2}}$ (*F*_1.93,24.81_ = 435.50, *P* < 0.0001) where $F_{IO_{2}}$ and $S_{pO_{2}}$ were significantly reduced immediately post‐titration (*P* < 0.0001) as well as 1 h (*P* < 0.0001) and 2 h (*P* < 0.0001) post‐titration (Figure 1). Clamping $F_{IO_{2}}$ resulted in greater levels of $S_{pO_{2}}$ compared to the protocol in which $S_{pO_{2}}$ was clamped (main effect of protocol: *F*_1,18_ = 109.80, *P* < 0.0001), whereas the protocol in which $S_{pO_{2}}$ was clamped resulted in a greater reduction in $F_{IO_{2}}$, compared to the protocol in which $F_{IO_{2}}$ was clamped (main effect of protocol: *F*_1,18_ = 7.72, *P* = 0.012). Furthermore, a protocol by time interaction effect was detected for $F_{IO_{2}}$ (*F*_4,72_ = 6.44, *P* = 0.001) and $S_{pO_{2}}$ (*F*_4,72_ = 30.32, *P* < 0.0001), where *post hoc* analysis revealed the protocol in which $S_{pO_{2}}$ was clamped caused a significantly greater reduction in $F_{IO_{2}}$ immediately post‐titration compared to the protocol in which $F_{IO_{2}}$ was clamped (*P* = 0.002), whereas the protocol in which $S_{pO_{2}}$ was clamped caused a significantly greater reduction in $S_{pO_{2}}$ immediately post‐titration ($F_{IO_{2}}$ clamping protocol: 87.5 ± 0.7%, $S_{pO_{2}}$ clamping protocol: 81.9 ± 1.3%; *P* < 0.0001) as well as 1 h ($F_{IO_{2}}$ clamping protocol: 87.4 ± 2.0%, $S_{pO_{2}}$ clamping protocol: 82.2 ± 1.5%; *P* < 0.0001) and 2 h ($F_{IO_{2}}$ clamping protocol: 90.6 ± 2.5%, $S_{pO_{2}}$ clamping protocol: 81.8 ± 2.7%; *P* < 0.0001) post‐titration compared to the protocol in which $F_{IO_{2}}$was clamped.

### 3.2 Symptoms of hypoxia

The Lake Louise AMS survey was used to assess subjective symptoms of hypoxia. A main effect of time was identified for AMS scores (main effect of time: *F*_2.27,40.90_ = 5.16, *P* = 0.008), where AMS scores significantly increased from baseline at 2 h (*P* = 0.011) post‐titration (Table 2). Changes in AMS were not associated with the protocol that the participants underwent (no main effect of protocol: *F*_1,18_ = 0.052, *P* = 0.822; no protocol by time interaction: *F*_4,72_ = 0.780, *P* = 0.542).

**TABLE 2:** Lake Louise Acute Mountain Sickness survey accumulative score.

	$F_{IO_{2}}$ clamping protocol (*n* = 10)	$S_{pO_{2}}$ clamping protocol (*n* = 10)
Baseline	0.10 ± 0.32	0.05 ± 0.16
Post‐titration	0.35 ± 0.41	0.15 ± 0.24
1 h	0.35 ± 0.69	0.25 ± 0.35
2 h	0.55 ± 0.69^#^	0.55 ± 0.60^#^
Reoxygenation	0	0.2 ± 0.48

All data are presented as means ± SD. Maximal AMS score is 12. #Significant difference from baseline scores (*P* = 0.011). $F_{IO_{2}}$, fraction of inspired oxygenation; $S_{pO_{2}}$, peripheral capillary oxygen saturation.

### 3.3 F‐wave responses

**FIGURE 2:**
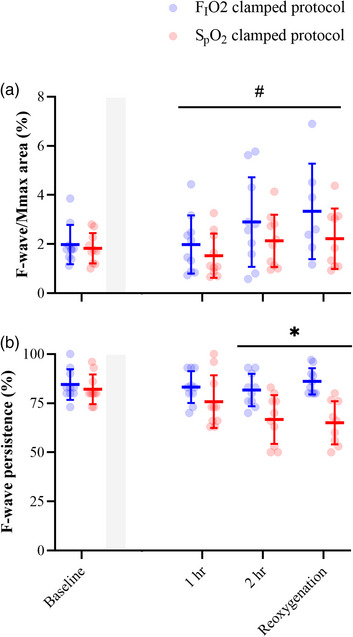
F‐wave area (a) and F‐wave persistence (b). Excitability of spinal motoneurones was assessed by performing supramaximal electrical stimulation of the ulna nerve, and then extracting F‐waves from FDI EMG. Measurements were made at baseline prior to each protocol, and then immediately post‐titration, 1 h post‐titration, 2 h post‐titration, and at reoxygenation after the removal of the hypoxaemic stimulus. Shaded area represents titration phase. Blue represents the $F_{IO_{2}}$ clamping protocol while red represents the $S_{pO_{2}}$ clamping protocol. #Significant *post hoc* analysis from main effect of time for F‐wave area (*P* = 0.037). *Significant effect of clamping arising from *post hoc* analysis of the interaction effect for F‐wave persistence at 2 h (*P* = 0.023) and following reoxygenation (*P* = 0.001). Data are presented as the group mean and error bars represent the standard deviation (*n* = 10 participants for all variables).

F‐wave responses were extracted from all participants for each time point in both the $F_{IO_{2}}$ and $S_{pO_{2}}$ clamping protocols. Although the amplitude of F‐waves progressively increased throughout hypoxic exposure compared to baseline measurements (main effect of time: *F*_2.14,35.68_ = 3.54, *P* = 0.037; Figure 2a), there were no differences between protocols (no main effect of protocol: *F*_1,18_ = 1.96, *P* = 0.179; no protocol by time interaction: *F*_3,50_ = 0.54, *P* = 0.654). With respect to the persistence of F‐waves, there was also a progressive decrease from baseline throughout hypoxic exposure (main effect of time: *F*_2.50,44.15_ = 6.394, *P* = 0.002; Figure 2b). However, in contrast to F‐wave amplitude, the persistence of F‐waves was lower during the $S_{pO_{2}}$ clamping protocol compared to the $F_{IO_{2}}$ protocol (interaction effect: *F*_3,53_ = 5.863, *P* = 0.002) at 2 h post‐titration (*P* = 0.023) and following reoxygenation (*P* = 0.001).

### 3.4 Unconditioned MEP responses

**FIGURE 3:**
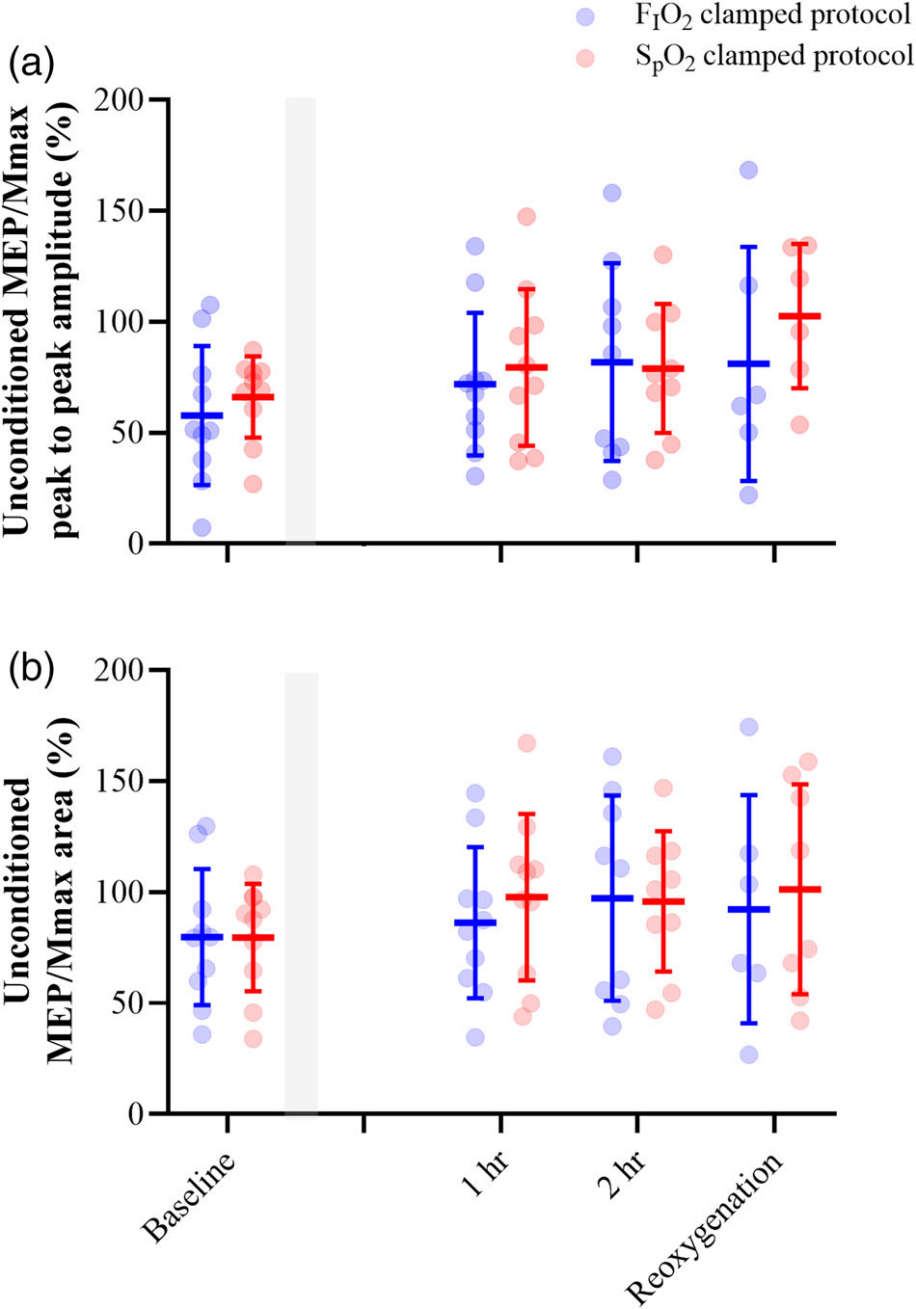
Unconditioned MEP responses. Excitability of the corticospinal tract was assessed throughout both protocols. The peak to peak amplitude (a) and area (b) of the MEP responses did not differ throughout, or between, the hypoxaemic‐inducing protocols. Shaded area represents titration phase. Blue represents the $F_{IO_{2}}$ clamping protocol while red represents the $S_{pO_{2}}$ clamping protocol. Data are presented as the group mean and error bars represent the standard deviation (*n* = 10 participants for all variables).

Throughout both protocols, corticospinal excitability remained consistent, as there was neither a main effect of time or protocol nor an interaction, for unconditioned MEP/*M*_max_ peak to peak amplitude (*F* < 3.0, *P >* 0.12; Figure 3a) and area (*F* < 2.4, *P* > 0.09; Figure 3b). Specifically, unconditioned MEP/*M*_max_ peak to peak amplitude was comparable at baseline ($F_{IO_{2}}$ protocol: 58 ± 31%, $S_{pO_{2}}$ protocol: 66 ± 18%), 1 h ($F_{IO_{2}}$ protocol: 72 ± 32%, $S_{pO_{2}}$ protocol: 79 ± 35%), 2 h ($F_{IO_{2}}$ protocol: 82 ± 45%, $S_{pO_{2}}$ protocol: 79 ± 32%) and reoxygenation ($F_{IO_{2}}$ protocol: 81 ± 53%, $S_{pO_{2}}$ protocol:102 ± 33%). Unconditioned MEP/*M*_max_ area was comparable at baseline ($F_{IO_{2}}$ protocol: 80 ± 30%, $S_{pO_{2}}$ protocol: 80 ± 24%), 1 h ($F_{IO_{2}}$ protocol: 86 ± 34%, $S_{pO_{2}}$ protocol: 98 ± 38%), 2 h ($F_{IO_{2}}$ protocol: 97 ± 46%, $S_{pO_{2}}$ protocol: 96 ± 32%) and reoxygenation ($F_{IO_{2}}$ protocol: 92 ± 51%, $S_{pO_{2}}$ protocol: 101 ± 47%).

### 3.5 Conditioned MEP responses

**FIGURE 4:**
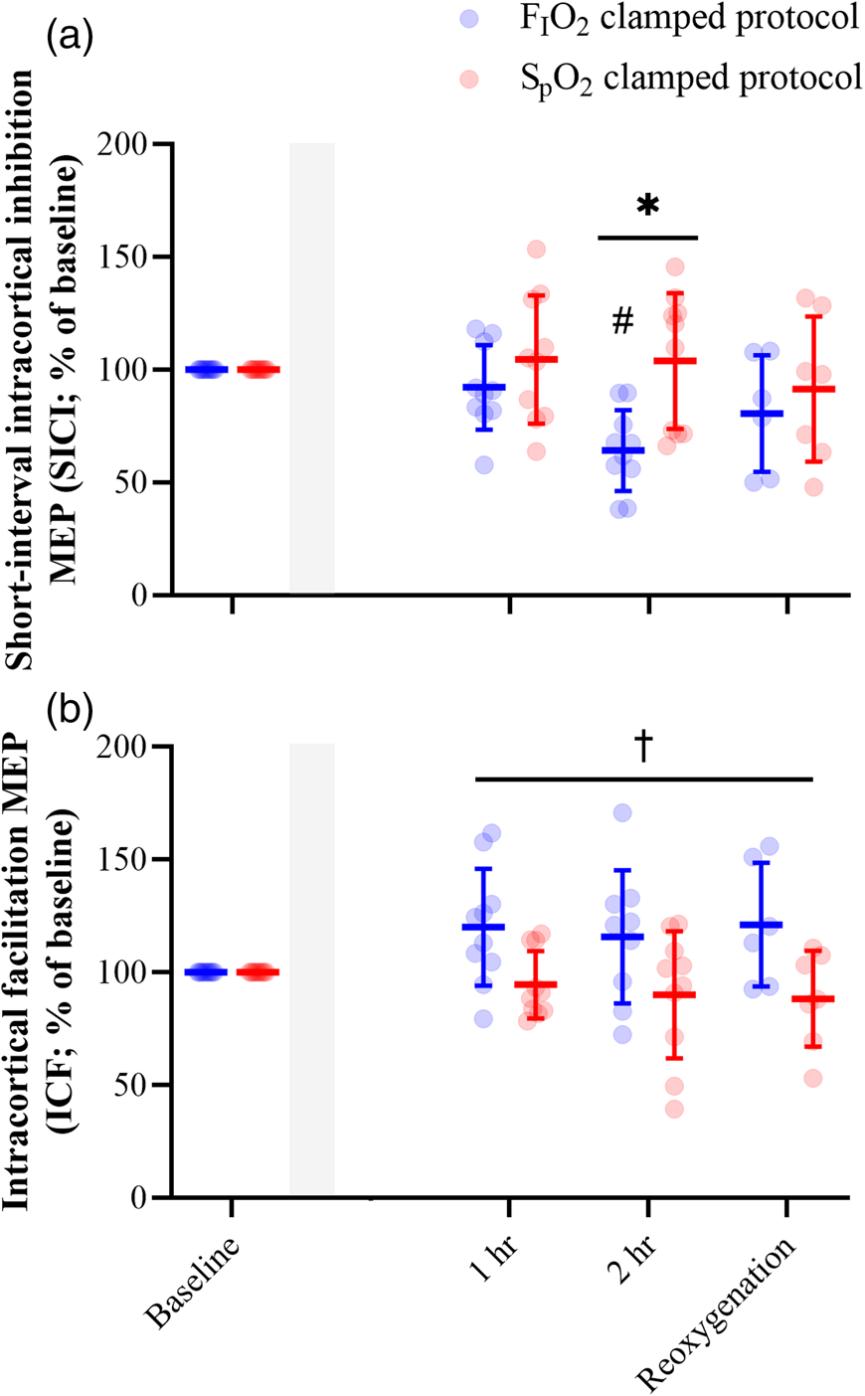
Conditioned MEP responses. Short‐interval intracortical inhibition (SICI; a) and intracortical facilitation (ICF; b) responses were assessed using paired‐pulse TMS paradigms. Normalised SICI and ICF MEP responses are expressed as percentage changes from baseline where a response >100% indicates less inhibition and greater facilitation of the MEP, while a response <100% indicates greater inhibition and less facilitation of the MEP, respectively. Shaded area represents titration phase where the $F_{IO_{2}}$ and $S_{pO_{2}}$ clamping targets were achieved. Blue represents the $F_{IO_{2}}$ clamping protocol while red represents the $S_{pO_{2}}$ clamping protocol. *Significant effect of clamping arising from *post hoc* analysis of the interaction effect at 2 h (*P* = 0.011). #Significant *post hoc* analysis from main effect of time for SICI at 2 h (*P* = 0.014). Cross represents significant main effect of clamping for ICF (*P* = 0.005). Data are presented as the group mean and error bars represent the standard deviation (*n* = 10 participants for all variables).

Paired‐pulse TMS paradigms were employed to assess motor cortical inhibition and facilitation. A main effect of time (*F*_2.36,36.97_ = 4.48, *P* = 0.014), main effect of protocol (*F*_1,18_ = 4.616, *P* = 0.046), and interaction effect (*F*_3,47_ = 4.916, *P* = 0.005) were determined during the SICI paradigm (Figure 4a). *Post hoc* analysis on the interaction effect indicated that the SICI MEP during the $F_{IO_{2}}$ clamping protocol was smaller than the SICI MEP during the $S_{pO_{2}}$ clamping protocol at 2 h (*P* = 0.011). With respect to ICF, a main effect of protocol (*F*_1,18_ = 9.972, *P* = 0.005), and an interaction effect (*F*_3,46_ = 3.269, *P* = 0.029) was detected. However, *post hoc* analysis on the interaction effect did not reach statistical significance at any particular time point (*P* > 0.05).

### 3.6 Relationship between conditioned MEP responses and blood oxygenation

**FIGURE 5:**
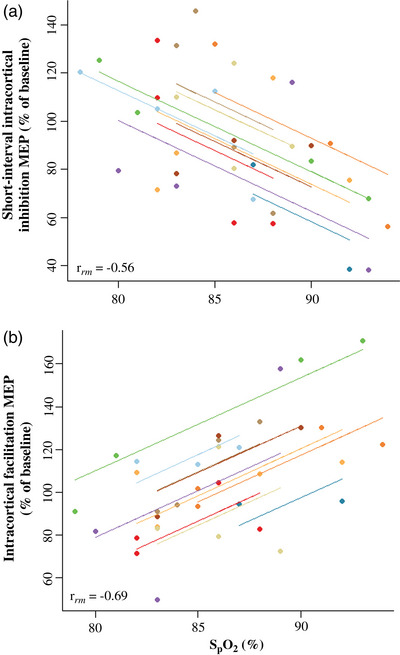
Relationship between conditioned MEP responses and blood oxygenation. The two clamping protocols resulted in distinct changes in $S_{pO_{2}}$ for each participant and a range of hypoxaemic severities. Consequently, data from both protocols were pooled together according to $S_{pO_{2}}$. Each colour represents the correlation for each participant. A negative correlation between SICI and $S_{pO_{2}}$ (*r*_rm_ = −0.56) and a positive correlation with ICF and $S_{pO_{2}}$ (*r*_rm_ = 0.69) were determined, where reductions in $S_{pO_{2}}$ resulted in less inhibition and less facilitation of MEP responses, respectively (*n* = 10 participants for all variables).

The two clamping protocols resulted in heterogeneous responses in $S_{pO_{2}}$ for each participant (Figure 1b). MEP data from both clamping protocols were pooled together according to $S_{pO_{2}}$ and repeated measures correlations coefficients were used to assess the relationship between SICI and ICF MEPs with $S_{pO_{2}}$ (Figure 5). The correlations indicated that the lower the $S_{pO_{2}}$, the less inhibition on the MEP during SICI‐inducing stimulations (*r*_rm_ (25) = −0.56, *P* = 0.002, CI = −0.77 to −0.23; Figure 5a) and the less facilitation of the MEP during ICF‐inducing stimulations (*r*_rm_ (24) = 0.69, *P* = 0.001, CI = 0.42–0.85; Figure 5b).

## 4 DISCUSSION

The purpose of this study was to examine how two acute continuous hypoxia protocols impact GABAergic and glutamatergic circuits responsible for inhibition (SICI) and facilitation (ICF) of corticospinal excitability, respectively, as well as spinal excitability. A hypoxic stimulus was employed to a $F_{IO_{2}}$ target of 0.13 and a $S_{pO_{2}}$ target of 80%. The main findings were (1) after an initial reduction, peripheral capillary oxygen saturation ($S_{pO_{2}}$) during $F_{IO_{2}}$ clamping began to return to baseline levels throughout the 2 h exposure; (2) the persistence of F‐wave responses was significantly reduced 2 h post‐titration and following reoxygenation for the $S_{pO_{2}}$ clamping protocol but not the $F_{IO_{2}}$ clamping protocol; (3) MEP responses during SICI in the $F_{IO_{2}}$ clamping protocol were smaller than baseline and the $S_{pO_{2}}$ clamping protocol at 2 h post‐titration, while MEP responses during ICF in the $F_{IO_{2}}$ clamping protocol were larger than in the $S_{pO_{2}}$ clamping protocol; and (4) correlative relationships between SICI, ICF and $S_{pO_{2}}$ suggest the degree of inhibition and facilitation on MEP responses may be impacted by the severity of hypoxaemia.

### 4.1 Level of hypoxaemia differs between $F_{IO_{2}}$ and $S_{pO_{2}}$ clamping

During both hypoxaemia protocols, $S_{pO_{2}}$ was reduced during a 15 min titration phase. Although this resulted in a reduction in $S_{pO_{2}}$ immediately after the titration phase, the $S_{pO_{2}}$ during the $F_{IO_{2}}$ clamping protocol was significantly greater than during the $S_{pO_{2}}$ clamping protocol. Furthermore, $S_{pO_{2}}$ during the $F_{IO_{2}}$ clamping protocol progressively increased throughout the 2 h duration whereas $S_{pO_{2}}$ remained lower during the $S_{pO_{2}}$ clamping protocol. At a given simulation of high altitude, oxygen availability is largely defended by an increase in ventilation (hypoxic ventilatory response), heart rate and oxygen offloading to tissue (Schoene et al., 1990; Sutton et al., 1992). This is due to increased activation of peripheral chemoreceptors, mechanoreceptors within the lungs, and further augmentation by central chemoreceptors in the brainstem (Schoene et al., 1990). These responses are likely to have occurred throughout the 2 h exposure to hypoxaemia in the $F_{IO_{2}}$ clamping protocol (Bird et al., 2021). However, hypocapnia can occur in conjunction with changes in hyperventilation and is a potent modulator of cerebral blood flow resulting in reduced cerebral oxygenation (Ainslie & Poulin, 2004; Rupp et al., 2015). Indeed, cerebral oxygenation at rest is markedly reduced when assessed using near‐infrared spectroscopy (Ruggiero et al., 2018; Rupp et al., 2015) and middle cerebral artery velocity (Ainslie et al., 2007). However, muscle oxygenation is maintained. As oxygenation in the current study was derived from pulse oximetry of the finger, we acknowledge that the increase in peripheral oxygen saturation observed during the $F_{IO_{2}}$ clamping protocol is not reflective of comparable compensation in cerebral oxygen saturation and a reduction in the resultant neural tissue hypoxia. As documented in this study and discussed in further detail, impairment in intracortical motor circuits did occur in the presence of improving $S_{pO_{2}}$, which suggests, in part, that a reduction in cortical oxygenation may have been maintained.

Considering this, our study demonstrates that the traditional method of inducing hypoxia in a laboratory setting by titrating either the input of the air stimulus to the system (changes in the $F_{IO_{2}}$) or the output of the system in response to the hypoxic stimulus (changes in the $S_{pO_{2}}$; Costello et al., 2020; Rojas‐Camayo et al., 2018) can result in progressively different exposures to tissue hypoxia. Taking this into account, future hypoxic research needs to consider these methodological differences.

### 4.2 Corticospinal and spinal motoneurone excitability during hypoxia

In the current study, MEP responses in the FDI remained consistent between clamping protocols and baseline measures obtained prior to each protocol. This signifies that an acute time course of hypoxic exposure did not impair corticospinal excitability. Indeed, previous accounts of acute hypoxic exposure for a similar duration (<2 h) have had little effect on MEP responses in the biceps brachii, FDI and quadriceps (Goodall et al., 2012; McKeown et al., 2022; Millet et al., 2012; Szubski et al., 2006). However, changes in other parameters of corticospinal excitability have been reported (i.e., decreased resting motor thresholds and shortening of EMG silent period; Szubski et al., 2006). MEP and motor threshold responses are dependent on the membrane excitability of corticospinal neurones as well as other cortical and spinal circuits above and below the motor cortex responsible for motor output (Ziemann et al., 1996), all of which have been reported to be impacted by hypoxia. While the method for delivering a hypoxic stimulus has varied across previous experiments, resulting in varied exposures to hypoxaemia, the current study employed tightly controlled titrations for each hypoxic stimulus. Considering this, the similarity in corticospinal excitability between both conditions may be attributed to the neural tissue experiencing iso‐hypoxaemia.

By eliciting F‐wave responses in the FDI we are able to provide insight into the excitability of a subpopulation of higher threshold α‐motoneurones without the input of descending drive from the motor cortex (Espiritu et al., 2003; Rothwell et al., 2021). In both hypoxia protocols, F‐wave amplitude progressively increased and remained so once oxygen delivery was returned to normoxic levels. However, during the $S_{pO_{2}}$ clamping protocol, F‐wave persistence progressively decreased and was significantly lower than responses during the $F_{IO_{2}}$ clamping protocol at 2 h and once returning to normoxia. Contrary to the current findings, in previous assessments, spinal excitability in intermittent and continuous hypoxaemia has remained unchanged when assessed indirectly (F‐wave responses; Christiansen et al., 2018; Miscio et al., 2009; Szubski et al., 2006) and directly without the influence of afferent input (cervicomedullary MEPs; Ruggiero et al., 2018). Interestingly, a 30 min intermittent exposure to hypoxaemia has demonstrated that increased output of FDI spinal motoneurones may be due to changes in the presynaptic excitability of corticospinal‐motoneuronal synapses (indicated during spike timing‐dependent plasticity stimulations), and not in α‐motoneurones directly (indicated during F‐wave responses to ulnar nerve stimulation; Christiansen et al., 2018). However, intermittent exposures to hypoxaemia have been identified to have distinct mechanisms of action on the CNS that differ from continuous exposures, those being ones facilitating plasticity in the corticospinal pathway, sympathetic nerve activity, as well as changing the connectivity of spinal interneurons, which may abolish the depression of α‐motoneurones excitability seen in continuous exposures to hypoxaemia (Christiansen et al., 2018; Dick et al., 2007; Streeter et al., 2017). Nonetheless, the presence of reduced F‐wave persistence in the $S_{pO_{2}}$ clamping protocol of the current study may be due to a greater degree of Ia afferents being activated during the supramaximal stimulation of the ulnar nerve. This, the presence of a greater hypoxic stimulus may have impacted the function of intrinsic voltage‐gated channels of the motoneurone (Cummins et al., 1993).

### 4.3 Short intracortical inhibition during hypoxia

Paired‐pulse TMS was used to assess changes in intracortical inhibition throughout the 2 h exposures to hypoxia and once reoxygenated following a return to normoxia. Intracortical inhibition is primarily mediated by the activity of GABAergic neurones; however, their activity can be influenced by other neurotransmitter systems. Differences in SICI responses between the two protocols were evident once 2 h of exposure had passed, where the MEP was inhibited to a greater extent during the $F_{IO_{2}}$ clamping protocol compared to the $S_{pO_{2}}$ clamping protocol. In line with our F‐wave findings, the ability of intracortical circuits to inhibit corticospinal excitability was reduced in the more severe exposure to hypoxaemia. This indicates that the impact of hypoxia on intracortical circuits responsible for inhibition of motor output may be severity‐ and time‐dependent. Indeed, MEP responses assessed in the resting FDI have demonstrated that more severe exposures to hypoxaemia for short durations have no effect on SICI compared to normoxia (75% $S_{aO_{2}}$ for <1 h; Szubski et al., 2006), while reductions in SICI have been demonstrated in less severe and longer durations of hypoxaemia (85% $S_{aO_{2}}$ for 3–5 days at high altitude; Miscio et al., 2009). This gives further support to the presence of a time and severity dependency for hypoxia's effect on inhibitory circuits. GABAergic neurones have a relatively high mitochondrial density compared to other neurones, indicating a greater reliance on oxygen availability, and they would likely be preferentially impacted by cerebral hypoxia (Sloper et al., 1980). Furthermore, animal studies have provided evidence that excessive activation of *N*‐methyl‐d‐aspartate (NMDA) receptors of GABAergic neurones occurs when hypoxic and results in neuronal degeneration (Romijn, 1989). Collectively, these findings suggest that hypoxia impacts the activity of GABAergic neurones; however, whether this is due to a direct mechanism as mentioned above or due to the indirect activity of other neurotransmitter systems cannot be determined in the current study.

### 4.4 Intracortical facilitation during hypoxia

ICF represents the excitability of facilitatory cortical, and potentially spinal, networks that are dependent on the activity of the glutamatergic system which overlies GABAergic inhibition (Hanajima et al., 1998; Wiegel et al., 2018; Ziemann et al., 2015). In the current study, ICF MEP responses during the $F_{IO_{2}}$ clamping protocol were greater than MEP responses during the $S_{pO_{2}}$ clamping protocol throughout the experimental protocol. This indicates that activity of facilitatory cortical and spinal circuits was less during the severe hypoxaemic stimulus where hypoxaemia exceeded 85% $S_{pO_{2}}$, but not the less severe stimulus. Activity of facilitatory circuits within the CNS is sensitive to changes in the activation of voltage‐dependent sodium channels (Liepert et al., 1997). As exposures to anoxia have been shown to inactivate these channels and reduce excitatory postsynaptic potentials (Cummins et al., 1993), the absence of facilitation in the more severe exposure to hypoxaemia in the current study may be due to reduced voltage‐dependent channel activation or reduced α‐motoneurone excitability similar to what was seen in our F‐wave assessment. However, previous assessments of ICF suggest that upon exposure to acute (Szubski et al., 2006) and chronic (Miscio et al., 2009) continuous hypoxaemia, glutamatergic neural circuits are not affected. This may be due to the compensatory effect of other excitatory neurotransmitter pathways. This indicates excitability of facilitatory networks can be maintained when hypoxic, which is likely dependent on the mode of hypoxaemia delivery, degree of tissue hypoxia and activity of other neuromodulatory pathways.

### 4.5 Excitability of intracortical networks is dependent on the duration and severity of hypoxaemia

Collectively, these findings provide evidence that the duration of exposure to hypoxaemia is important when determining the consequences of hypoxia on intracortical networks. However, as each individual experienced a varying degree of hypoxaemia during the $F_{IO_{2}}$ clamping protocol, we can distinguish if the activity of intracortical networks is also dependent on the severity of hypoxaemia. Indeed, we found that the activity of GABAergic neurones reduced as the severity of hypoxaemia increased. Likewise, the activity of glutamatergic neurones was affected by acute hypoxaemia in a similar manner to GABAergic neurones, where the degree of MEP facilitation decreases as the severity of hypoxaemia increases. Based on these findings, a hypoxic threshold for intracortical network activity to inhibit or facilitate motor output may exist at exposures <85% $S_{pO_{2}}$, where a significant amount of time below this threshold may result in the modulatory effect of these circuits being diminished. In chronic exposures to hypoxaemia, Miscio et al. (2009) found similar correlative relationships where the degree of SICI reduced as the severity of hypoxaemia increased to 84% of arterial oxygen saturation after 3–5 days at high‐altitude. However, we acknowledge that other physiological adaptations occur during chronic exposure to hypoxia which can also contribute to the reduction in neuronal excitability (respiratory alkalosis, chronic hyperventilation and sleep apnoea; Civardi et al., 2004; Leaf & Goldfarb, 2007). Nonetheless, this finding provides insight into the underlying mechanisms of cortical function when hypoxic and may explain why corticospinal tract responses differ in varying exposures to acute and chronic hypoxaemia (Rupp et al., 2012).

### 4.6 Considerations

The adaptations found in GABAergic and glutamatergic neurones may not have occurred in isolation. That is, hypoxia may have had an influence on other parameters of cortical and spinal circuit activity and other neuromodulator pathways (i.e., serotonergic and dopaminergic mechanisms) that were not assessed in the current study. Other parameters that influence the output of the motor cortex that can be assessed with paired‐pulse TMS consist of GABA‐B activity (long intracortical inhibition, LICI; McDonnell et al., 2006) and summation of I‐wave generation at corticospinal neurones (short‐interval intracortical facilitation, SICF; Tokimura et al., 1996). To date, the impact of hypoxia on LICI and SICF responses has not been examined and would provide further insight into the impact hypoxia has on CNS circuitry.

## 5 CONCLUSION

When exposed to two commonly used methods of exposure to hypoxaemia (0.13 $F_{IO_{2}}$ clamping protocol and 80% $S_{pO_{2}}$ clamping protocol), the ability of intracortical networks to inhibit and facilitate motor output is affected differently. This was demonstrated by a greater reduction in SICI responses and greater ICF responses during the $F_{IO_{2}}$ clamping protocol compared to the $S_{pO_{2}}$ clamping protocol.

## AUTHOR CONTRIBUTIONS

Data collection and analysis was performed by D.J.M. at Griffith University, Australia. All authors contributed to the conception and design of this work, as well as the drafting and final approval of the manuscript. All authors agree to be accountable for all aspects of the work in ensuring that questions related to the accuracy or integrity of any part of the work are appropriately investigated and resolved. All persons designated as authors qualify for authorship, and all those who qualify for authorship are listed.

## CONFLICT OF INTEREST

The authors declare no conflicts of interest.

## Data Availability

The data supporting the findings of this study are available from the corresponding author upon reasonable request.
